# 4-Cyano-1-methyl­pyridinium iodide

**DOI:** 10.1107/S1600536812032230

**Published:** 2012-07-21

**Authors:** Michael N. Kammer, Lynn V. Koplitz, Joel T. Mague

**Affiliations:** a Department of Physics, Loyola University, New Orleans, LA 70118, USA; b Department of Chemistry, Loyola University, New Orleans, LA 70118, USA; c Department of Chemistry, Tulane University, New Orleans, LA 70118, USA

## Abstract

In the crystal structure of the title compound, C_7_H_7_N_2_^+^·I^−^, the cations form inversion-related dimers *via* weak pairwise C—H⋯N hydrogen bonds. In the dimers, the pyridinium rings are parallel to one another with their mean planes separated by a normal distance of *ca* 0.28 Å. Weak C—H⋯N inter­actions between adjacent dimers generate a layer lying parallel to (10-1). The remaining H atoms form C—H⋯I inter­actions, which link the layers into a three-dimensional structure.

## Related literature

For the structure of 3-cyano-1-methyl­pyridinium iodide, see: Koplitz *et al.* (2003 ▶). For the structure of 1-methyl­pyridinium iodide, see: Lalancette *et al.* (1978 ▶). For related structures see: Mague *et al.* (2005 ▶); Koplitz *et al.* (2012 ▶). 
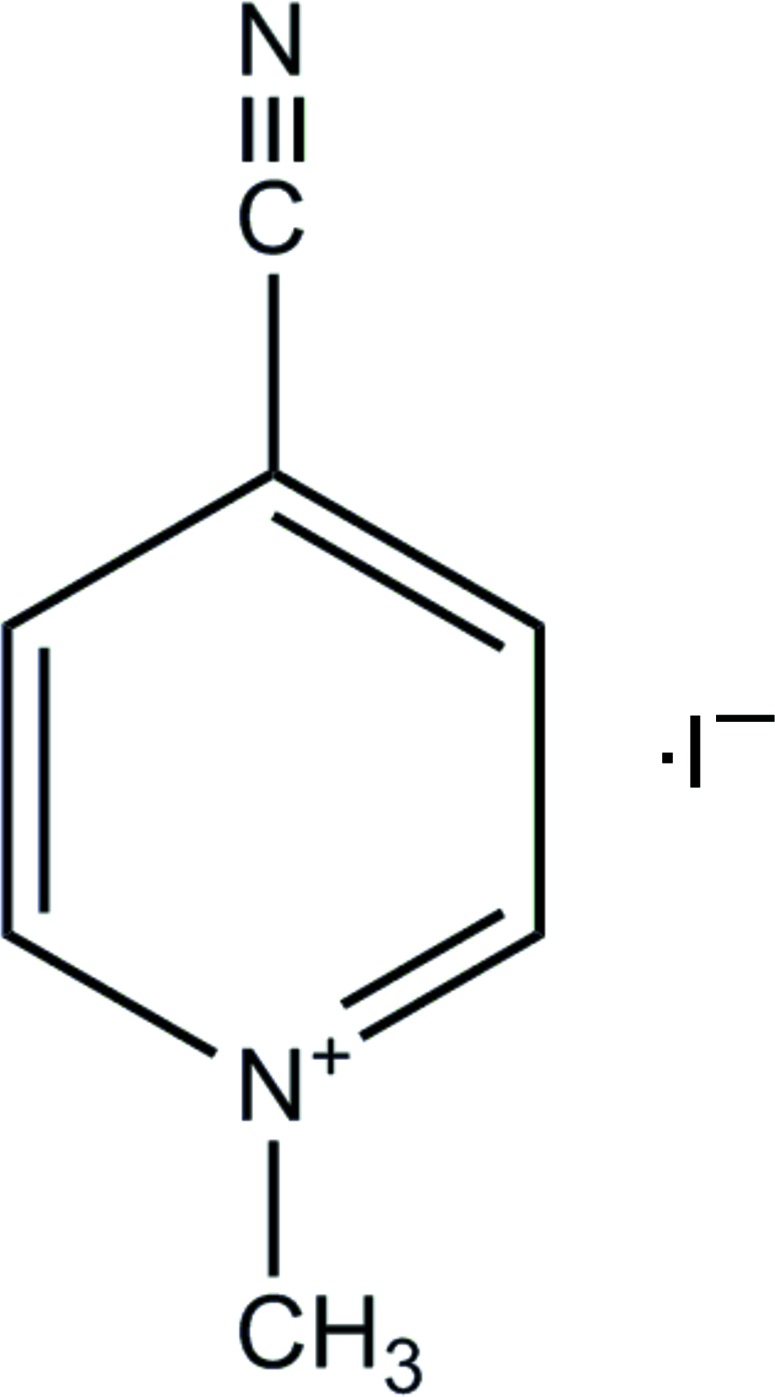


## Experimental

### 

#### Crystal data

C_7_H_7_N_2_^+^·I^−^
*M* *_r_* = 246.05
Monoclinic, 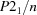

*a* = 5.0734 (3) Å
*b* = 11.4528 (7) Å
*c* = 15.0751 (9) Å
β = 99.679 (1)°
*V* = 863.46 (9) Å^3^
*Z* = 4
Mo *K*α radiation
μ = 3.64 mm^−1^
*T* = 100 K
0.14 × 0.07 × 0.05 mm

#### Data collection

Bruker SMART APEX CCD diffractometer
Absorption correction: multi-scan (*SADABS*; Sheldrick, 1996 ▶) *T*_min_ = 0.614, *T*_max_ = 0.836
12786 measured reflections
1792 independent reflections
1572 reflections with *I* > 2σ(*I*)
*R*_int_ = 0.040

#### Refinement

*R*[*F* ^2^ > 2σ(*F* ^2^)] = 0.020
*wR*(*F* ^2^) = 0.048
*S* = 1.07
1792 reflections
92 parameters
H-atom parameters constrained
Δρ_max_ = 0.88 e Å^−3^
Δρ_min_ = −0.47 e Å^−3^

### 

Data collection: *APEX2* (Bruker, 2010 ▶); cell refinement: *SAINT* (Bruker, 2009 ▶); data reduction: *SAINT*; program(s) used to solve structure: *SHELXS97* (Sheldrick, 2008 ▶); program(s) used to refine structure: *SHELXL97* (Sheldrick, 2008 ▶); molecular graphics: *SHELXTL* (Sheldrick, 2008 ▶); software used to prepare material for publication: *SHELXTL*.

## Supplementary Material

Crystal structure: contains datablock(s) I, global. DOI: 10.1107/S1600536812032230/su2473sup1.cif

Structure factors: contains datablock(s) I. DOI: 10.1107/S1600536812032230/su2473Isup2.hkl

Supplementary material file. DOI: 10.1107/S1600536812032230/su2473Isup3.cml

Additional supplementary materials:  crystallographic information; 3D view; checkCIF report

## Figures and Tables

**Table 1:** Hydrogen-bond geometry (Å, °)

*D*—H⋯*A*	*D*—H	H⋯*A*	*D*⋯*A*	*D*—H⋯*A*
C3—H3⋯N2^i^	0.95	2.58	3.434 (4)	149
C1—H1*B*⋯N2^ii^	0.98	2.71	3.513 (4)	140
C1—H1*A*⋯I1^iii^	0.98	3.04	3.999 (3)	166
C1—H1*C*⋯I1^iv^	0.98	3.06	3.870 (3)	141
C2—H2⋯I1^v^	0.95	2.99	3.796 (3)	144
C5—H5⋯I1^vi^	0.95	2.94	3.839 (3)	158
C6—H6⋯I1^iii^	0.95	3.01	3.916 (3)	161

Symmetry codes: (i) 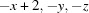; (ii) 
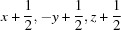
; (iii) 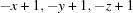; (iv) 
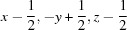
; (v) 
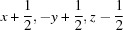
; (vi) 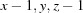.

**Fig. 1.**
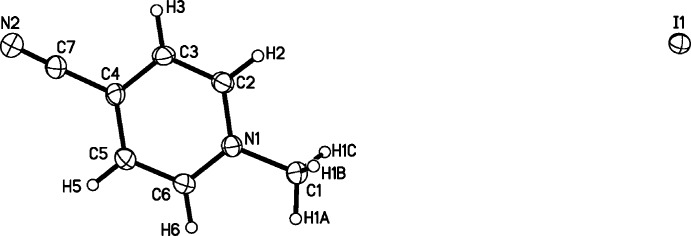
A perspective view of the asymmetric unit of the title compound with atom numbering. Displacement ellipsoids are drawn at the 50% probability level.

**Fig. 2.**
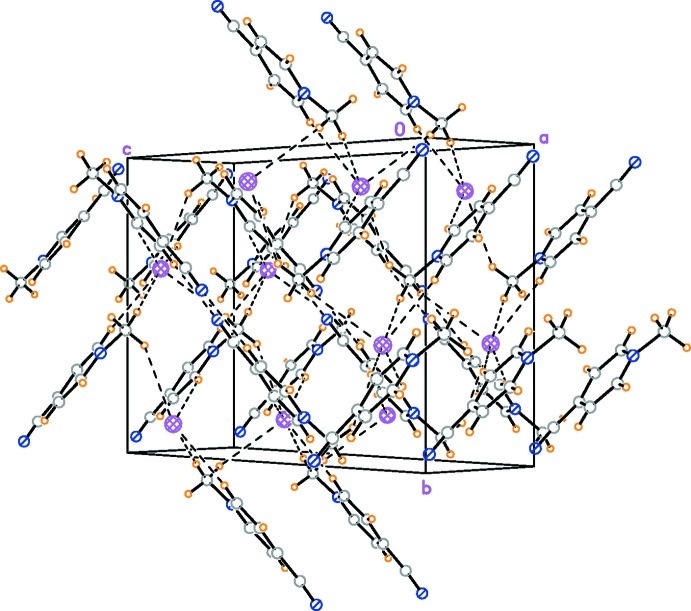
A view of the crystal packing of the title compound, showing the interpenetrating sheets of cations [colour key: C = gray, H = orange, N = blue, I = purple; C—H···I interactions are depicted as dashed lines].

## supplementary crystallographic information

### Comment

Previously reported structures of four other cyano-1-methylpyridinium salts (Koplitz *et al.*, 2003; Mague *et al.*, 2005; Koplitz *et al.*, 2012) include three layered compounds with all atoms, except the methyl H atoms, lying on crystallographic mirror planes. Interestingly, none of the iodide salts of the 4-, 3- and 2-cyano-1-methylpyridinium cation adopt this layer structure, possibly because the larger size and weaker hydrogen-bonding ability of iodide as compared with the smaller chloride and bromide ions provides a less restrictive set of interionic interactions.

The molecular structure of the title compound is illustrated in Fig. 1. In the crystal, the cations form inversion dimers *via* weak pairwise C2—H2···N2 hydrogen bonds (Table 1). In the dimers the pyridinium rings are parallel to one another with their mean planes separated by a normal distance of *ca* 0.28 Å. Weak C1—H1B···N2 interactions between adjacent dimers generate a layer lying parallel to (101), with the remaining hydrogen atoms forming C—H···I interactions (Table 1). The latter reinforce the construction of the layers as well as tying them together into a three-dimensional structure (Fig. 2).

In contrast to 3-cyano-1-methylpyridinium iodide (Koplitz *et al.*, 2003) where each iodide ion interacts with three C—H groups, in the title compound each anion is linked by five C—H groups which may reflect the more linear shape of the cation in the present structure.

### Experimental

4-Cyanopyridine (10.55 g) was dissolved in benzene (40 ml). Iodomethane (9.5 ml) was added to this solution slowly with stirring and the solution was refluxed for 75 minutes. A yellow solid was collected by vacuum filtration (*M*.p. 462 - 466 K). Addition of ethanol to the supernatant (*ca* 2:1 benzene:ethanol) resulted in the the growth overnight of thin plate-like yellow crystals of the title compound, suitable for X-ray diffraction.

### Refinement

The C-bound H-atoms were included in calculated positions and treated as riding atoms: C—H = 0.95 and 0.98 Å for CH and CH_3_ H-atoms, respectively, with *U*_iso_(H) = k × *U*_eq_(C), where k = 1.5 for CH_3_ H-atoms and 1.2 for other H-atoms.

### Crystal data

**Table:**

C_7_H_7_N_2_^+^·I^−^	*F*(000) = 464
*M**_r_* = 246.05	*D*_x_ = 1.893 Mg m^−^^3^
Monoclinic, *P*2_1_/*n*	Mo *K*α radiation, λ = 0.71073 Å
Hall symbol: -P 2yn	Cell parameters from 8899 reflections
*a* = 5.0734 (3) Å	θ = 2.3–28.6°
*b* = 11.4528 (7) Å	µ = 3.64 mm^−^^1^
*c* = 15.0751 (9) Å	*T* = 100 K
β = 99.679 (1)°	Plates, yellow
*V* = 863.46 (9) Å^3^	0.14 × 0.07 × 0.05 mm
*Z* = 4	

### Data collection

**Table:**

Bruker SMART APEX CCD diffractometer	1792 independent reflections
Radiation source: fine-focus sealed tube	1572 reflections with *I* > 2σ(*I*)
Graphite monochromator	*R*_int_ = 0.040
φ and ω scans	θ_max_ = 26.5°, θ_min_ = 2.3°
Absorption correction: multi-scan (*SADABS*; Sheldrick, 1996)	*h* = −6→6
*T*_min_ = 0.614, *T*_max_ = 0.836	*k* = −14→14
12786 measured reflections	*l* = −18→18

### Refinement

**Table:**

Refinement on *F*^2^	Primary atom site location: structure-invariant direct methods
Least-squares matrix: full	Secondary atom site location: difference Fourier map
*R*[*F*^2^ > 2σ(*F*^2^)] = 0.020	Hydrogen site location: inferred from neighbouring sites
*wR*(*F*^2^) = 0.048	H-atom parameters constrained
*S* = 1.07	*w* = 1/[σ^2^(*F*_o_^2^) + (0.0159*P*)^2^ + 1.1195*P*] where *P* = (*F*_o_^2^ + 2*F*_c_^2^)/3
1792 reflections	(Δ/σ)_max_ = 0.002
92 parameters	Δρ_max_ = 0.88 e Å^−^^3^
0 restraints	Δρ_min_ = −0.47 e Å^−^^3^

### Special details

**Table:**

Geometry. All e.s.d.'s (except the e.s.d. in the dihedral angle between two l.s. planes) are estimated using the full covariance matrix. The cell e.s.d.'s are taken into account individually in the estimation of e.s.d.'s in distances, angles and torsion angles; correlations between e.s.d.'s in cell parameters are only used when they are defined by crystal symmetry. An approximate (isotropic) treatment of cell e.s.d.'s is used for estimating e.s.d.'s involving l.s. planes.
Refinement. H-atoms were placed in calculated positions (C—H = 0.95 - 0.98 Å) and included as riding contributions with isotropic displacement parameters 1.2 - 1.5 times those of the attached carbon atoms.

### Fractional atomic coordinates and isotropic or equivalent isotropic displacement parameters (Å^2^)

**Table:**

	*x*	*y*	*z*	*U*_iso_*/*U*_eq_	
I1	0.95185 (3)	0.378404 (15)	0.854589 (12)	0.02114 (7)	
N1	0.6792 (5)	0.3458 (2)	0.18850 (15)	0.0201 (5)	
N2	0.7382 (5)	0.0466 (2)	−0.07663 (17)	0.0307 (6)	
C1	0.6477 (6)	0.4209 (3)	0.26587 (19)	0.0233 (6)	
H1A	0.5052	0.4779	0.2472	0.035*	
H1B	0.8159	0.4621	0.2871	0.035*	
H1C	0.6013	0.3726	0.3146	0.035*	
C2	0.8704 (6)	0.2626 (3)	0.19989 (19)	0.0218 (6)	
H2	0.9858	0.2554	0.2562	0.026*	
C3	0.8996 (6)	0.1883 (3)	0.13096 (19)	0.0219 (6)	
H3	1.0361	0.1306	0.1387	0.026*	
C4	0.7265 (6)	0.1986 (2)	0.04961 (18)	0.0207 (6)	
C5	0.5356 (6)	0.2869 (3)	0.03797 (19)	0.0243 (6)	
H5	0.4201	0.2965	−0.0181	0.029*	
C6	0.5167 (6)	0.3604 (3)	0.10929 (19)	0.0223 (6)	
H6	0.3883	0.4215	0.1023	0.027*	
C7	0.7369 (6)	0.1158 (3)	−0.0225 (2)	0.0244 (6)	

### Atomic displacement parameters (Å^2^)

**Table:**

	*U*^11^	*U*^22^	*U*^33^	*U*^12^	*U*^13^	*U*^23^
I1	0.01891 (11)	0.02214 (12)	0.02205 (11)	0.00126 (7)	0.00256 (7)	0.00040 (7)
N1	0.0211 (12)	0.0212 (12)	0.0191 (12)	0.0000 (9)	0.0065 (9)	0.0021 (9)
N2	0.0343 (15)	0.0328 (15)	0.0257 (14)	0.0058 (12)	0.0071 (11)	−0.0015 (12)
C1	0.0265 (15)	0.0239 (15)	0.0201 (14)	0.0037 (12)	0.0055 (12)	0.0008 (11)
C2	0.0190 (14)	0.0260 (15)	0.0204 (14)	0.0032 (11)	0.0035 (11)	0.0053 (11)
C3	0.0197 (14)	0.0238 (15)	0.0237 (15)	0.0060 (11)	0.0078 (11)	0.0048 (11)
C4	0.0250 (15)	0.0217 (14)	0.0171 (14)	0.0001 (11)	0.0082 (11)	0.0021 (11)
C5	0.0229 (15)	0.0301 (17)	0.0190 (14)	0.0041 (12)	0.0010 (11)	0.0021 (12)
C6	0.0219 (14)	0.0228 (15)	0.0219 (14)	0.0053 (11)	0.0029 (11)	0.0033 (11)
C7	0.0253 (15)	0.0256 (16)	0.0234 (15)	0.0017 (12)	0.0073 (12)	0.0027 (12)

### Geometric parameters (Å, º)

**Table:**

N1—C6	1.343 (4)	C2—H2	0.9500
N1—C2	1.350 (4)	C3—C4	1.388 (4)
N1—C1	1.480 (4)	C3—H3	0.9500
N2—C7	1.139 (4)	C4—C5	1.390 (4)
C1—H1A	0.9800	C4—C7	1.451 (4)
C1—H1B	0.9800	C5—C6	1.381 (4)
C1—H1C	0.9800	C5—H5	0.9500
C2—C3	1.370 (4)	C6—H6	0.9500
			
C6—N1—C2	121.4 (2)	C2—C3—H3	120.5
C6—N1—C1	119.8 (2)	C4—C3—H3	120.5
C2—N1—C1	118.7 (2)	C3—C4—C5	119.8 (3)
N1—C1—H1A	109.5	C3—C4—C7	120.6 (3)
N1—C1—H1B	109.5	C5—C4—C7	119.5 (3)
H1A—C1—H1B	109.5	C6—C5—C4	118.8 (3)
N1—C1—H1C	109.5	C6—C5—H5	120.6
H1A—C1—H1C	109.5	C4—C5—H5	120.6
H1B—C1—H1C	109.5	N1—C6—C5	120.3 (3)
N1—C2—C3	120.6 (3)	N1—C6—H6	119.9
N1—C2—H2	119.7	C5—C6—H6	119.9
C3—C2—H2	119.7	N2—C7—C4	176.3 (3)
C2—C3—C4	119.0 (3)		
			
C6—N1—C2—C3	−1.6 (4)	C7—C4—C5—C6	175.8 (3)
C1—N1—C2—C3	177.5 (3)	C2—N1—C6—C5	2.4 (4)
N1—C2—C3—C4	−1.1 (4)	C1—N1—C6—C5	−176.7 (3)
C2—C3—C4—C5	2.9 (4)	C4—C5—C6—N1	−0.5 (4)
C2—C3—C4—C7	−175.0 (3)	C3—C4—C7—N2	76 (5)
C3—C4—C5—C6	−2.2 (4)	C5—C4—C7—N2	−102 (5)

### Hydrogen-bond geometry (Å, º)

**Table:**

*D*—H···*A*	*D*—H	H···*A*	*D*···*A*	*D*—H···*A*
C3—H3···N2^i^	0.95	2.58	3.434 (4)	149
C1—H1*B*···N2^ii^	0.98	2.71	3.513 (4)	140
C1—H1*A*···I1^iii^	0.98	3.04	3.999 (3)	166
C1—H1*C*···I1^iv^	0.98	3.06	3.870 (3)	141
C2—H2···I1^v^	0.95	2.99	3.796 (3)	144
C5—H5···I1^vi^	0.95	2.94	3.839 (3)	158
C6—H6···I1^iii^	0.95	3.01	3.916 (3)	161

Symmetry codes: (i) −*x*+2, −*y*, −*z*; (ii) *x*+1/2, −*y*+1/2, *z*+1/2; (iii) −*x*+1, −*y*+1, −*z*+1; (iv) *x*−1/2, −*y*+1/2, *z*−1/2; (v) *x*+1/2, −*y*+1/2, *z*−1/2; (vi) *x*−1, *y*, *z*−1.
